# Building a Multidisciplinary Cardiogenic Shock Program in Latin America: Outcomes From the TecSalud Program

**DOI:** 10.1016/j.jscai.2026.105508

**Published:** 2026-07-07

**Authors:** Vicente Jimenez-Franco, Jahir Rodriguez-Rivera, Renata Quevedo-Salazar, Jose A. Salinas-Casanova, Monica Flores-Zertuche, Cecilia Gocher-Janet, Juan Carlos Ibarrola-Peña, Carlos Jerjes-Sanchez, Rene D. Gomez-Gutierrez, Guillermo Quezada-Valenzuela, Erasmo de la Peña, Guillermo Torre-Amione

**Affiliations:** a Tecnológico de Monterrey, Escuela de Medicina y Ciencias de la Salud, Monterrey, Nuevo Leon, Mexico; b Instituto de Cardiología y Medicina Vascular, TecSalud, Escuela de Medicina y Ciencias de la Salud, Tecnológico de Monterrey, San Pedro Garza Garcia, Nuevo Leon, Mexico

**Keywords:** cardiogenic shock, low- and middle-income countries, mechanical circulatory support, multidisciplinary team, program development

## Abstract

**Background:**

Cardiogenic shock (CS) remains associated with high mortality despite modern reperfusion and mechanical circulatory support. Multidisciplinary “CS teams” have demonstrated improved outcomes; however, data from low- and middle-income countries are scarce. This study describes the experience and outcomes following the implementation of a dedicated CS team at a tertiary hospital in Mexico.

**Methods:**

We conducted a retrospective, single-center cohort study including consecutive adults with CS admitted to TecSalud between January 2021 and August 2025. The multidisciplinary CS team was established in August 2022. Patients were divided into 2 cohorts: pre-team (January 2021 to July 2022) and post-team (August 2022 to August 2025). The primary end point was 30-day all-cause mortality. Mortality rates were compared using the Fisher exact test.

**Results:**

A total of 67 patients were included (27 preteam, 40 postteam). Among the postteam cohort, the mean age was 63 ± 15.2 years, and 73% were male. The leading etiology was acute myocardial infarction–related CS (67.5%), followed by decompensated heart failure (20%). The median hospital stay was 11.5 days (0-122), and the average ICU stay was 7 days (1-105). Most patients presented in the Society for Cardiovascular Angiography & Interventions (SCAI) SHOCK stage E (33%), 80% required mechanical circulatory support, and 18% underwent extracorporeal cardiopulmonary resuscitation. Implementation of the CS team was associated with a reduction in 30-day mortality from 63% to 25%.

**Conclusions:**

The implementation of a multidisciplinary CS team at a tertiary center in Mexico was associated with a substantial reduction in 30-day mortality and improved care coordination. These findings support the feasibility and effectiveness of structured CS programs in complex health care environments.

## Introduction

Cardiogenic shock (CS) remains a fatal condition, with in-hospital mortality still hovering around 50% despite recent advances in mechanical circulatory support (MCS) and revascularization techniques.^1^^,^^2^ In recognition of this complexity of care—and the need to overcome the fragmentation often seen in traditional models—several institutions globally have developed multidisciplinary “CS teams,” comprising experts in critical care cardiology, advanced heart failure, interventional cardiology, and cardiac surgery. By promoting early recognition and coordinated care, shock teams have been associated with improved survival and more efficient use of resources.^3^

Although the impact of such teams shows promise, translating this model into low- and middle-income countries (LMIC) presents unique challenges. Persistently high mortality rates in these regions often reflect delayed recognition, limited access to MCS, and variability in institutional readiness.^4^^,^^5^ Moreover, the financial constraints and heterogeneity in training inherent to the region’s health systems can further hinder such teams' implementation and long-term sustainability.

On the other hand, hospital care in private hospitals across Latin America is primarily based on an open model of independent physician practices. In Mexico, there are approximately 3500 private hospitals across the country, almost all of which are private, for-profit, and with an open model of physician privileges. Establishing multidisciplinary teams focused on improving outcomes in a defined clinical setting is extraordinarily challenging under these conditions. TecSalud is a university-based health care system, a private and non-for-profit institution; within this framework, we have established several multidisciplinary programs aimed at improving outcomes in the hospital setting. To this end, we were faced with the recognition that the optimal care of CS patients was complex in nature and represented a clinical condition with high mortality, wherein new expensive technologies in the appropriate setting could result in major improvements in outcomes.

Recognizing these needs, our institution established the “TecSalud Cardiogenic Shock Program,” where we defined and adopted protocols from international models, creating a structured multidisciplinary collaboration that is often absent in open-model hospitals. This initiative included a deep institutional commitment to education, communication, and strategic resource allocation. This report outlines our experience in establishing a CS program and presents the clinical outcomes achieved after its implementation.

## Methods

**Figure 1:**
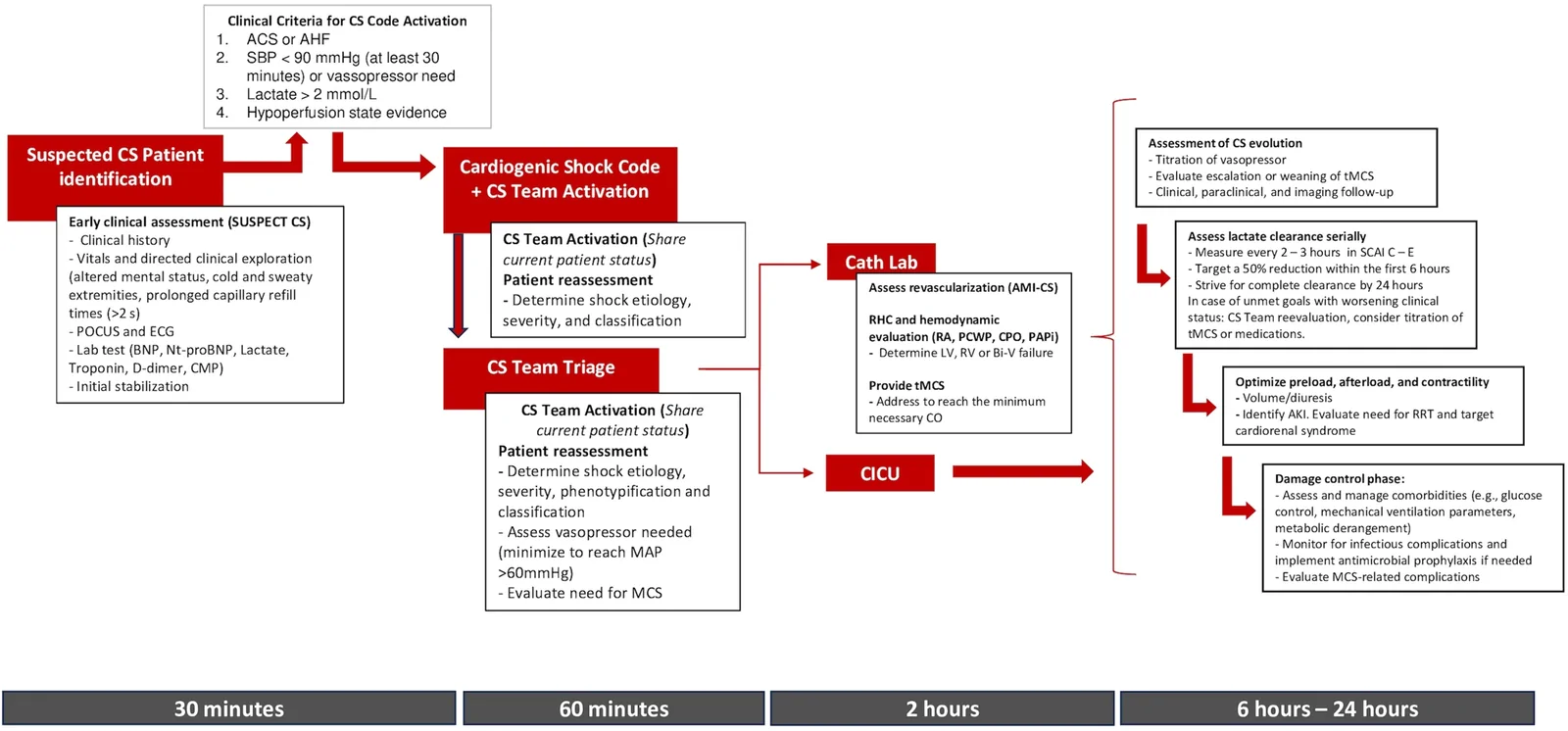
**TecSalud cardiogenic shock (CS) initial clinical pathway.** ACS, acute coronary syndrome; AHF, acute heart failure, AKI, acute kidney injury; AMI-CS, acute myocardial infarction–related cardiogenic shock; Bi-V, biventricular; BNP, brain natriuretic peptide; CMP, comprehensive metabolic panel; CO, cardiac output; CPO, cardiac power output; LV, left ventricle; MAP, mean arterial pressure; MCS, mechanical circulatory support; PAPI, pulmonary artery pulsatility index; POCUS, point-of-care ultrasound; PWCP, pulmonary wedge capillary pressure; RA, right atrium; RHC, right heart catheterization; RRT, renal replacement therapy; RV, right ventricle; SBP, systolic blood pressure; SCAI, Society for Cardiovascular Angiography & Interventions; tMCS, temporary mechanical circulatory support.

The TecSalud CS Program is an institutional initiative aimed at standardizing early recognition, optimizing clinical workflows, and improving outcomes through coordinated care for patients admitted with CS at Hospital Zambrano Hellion, the flagship hospital for the cardiovascular care center of TecSalud, part of Tecnológico de Monterrey, in Monterrey, Mexico. Following its implementation in August 2022, patients presenting with CS have been managed through a structured, multidisciplinary approach involving interventional cardiologists, cardiac surgeons, advanced heart failure specialists, critical care physicians, MCS specialists, and an institutional extracorporeal membrane oxygenation (ECMO) team. The initial phase included the development and activation of a standardized “CS Code,” which encompassed personnel education and training to facilitate early recognition and rapid response to CS across all health care providers. The evaluation and initial clinical approach of our institution is illustrated in Figure 1. This framework did not replace individual clinical judgment; rather, it established a structured, multidisciplinary governance model that reduced variability associated with isolated decision-making and ensured timely consensus among specialists. The algorithm functioned as a decision-support tool that prioritized early recognition, rapid escalation, and coordinated intervention, while still allowing flexibility based on patient-specific considerations.

### Study population and study design

We conducted a retrospective, single-center cohort study at TecSalud, a university-based health care system in Monterrey, Mexico. TecSalud is a tertiary center operating within a health care system characterized by significant regional resource disparities. Consecutive adult patients (≥18 years old) admitted with a diagnosis of CS between January 2021 and August 2025 were included in the registry. The diagnosis and staging of CS were defined according to the Society for Cardiovascular Angiography & Interventions (SCAI) criteria. Initial screening, inclusion criteria, and clinical care pathway are in Figure 1. Two distinct periods were analyzed:

• Pre-CS team era: January 2021 to July 2022, reflecting an 18-month period before the implementation of the multidisciplinary Shock Team.
• Post-CS team era: August 2022 to August 2025, 3 years after the establishment of the TecSalud CS Program.

The CS etiology was categorized into 3 primary groups: acute myocardial infarction–related CS, decompensated heart failure–related CS, and “other” etiologies. The “other” category encompasses postcardiotomy shock and procedurally related shock (eg, shock occurring in the immediate setting of cardiac surgery or high-risk structural interventions). and other types of cardiac dysfunction (eg, stress cardiomyopathy)

Patients during the post-CS era underwent longitudinal clinical surveillance as part of a quality improvement initiative, including routine outpatient follow-up and monitoring of any subsequent readmissions within the institutional system. Follow-up was continued until death or loss to follow-up. The clinical data of patients in the pre-CS era were retrospectively extracted from electronic medical records.

### Shock Team activation and evolution

The multidisciplinary CS team was intended for activation in all cases of suspected or confirmed CS. During the program’s inception, clinical recognition was initially most consistent in patients presenting with overt, critically ill phenotypes (SCAI stages D and E). As the clinical care pathway matured and institutional education increased, there was a significant shift toward the early identification of CS, allowing for team activation in less severe or “evolving” stages. This evolution reflects a broader institutional transition toward proactive, multidisciplinary management across various cardiovascular pathologies.

### Outcome

The primary objective of this study was to compare 30-day all-cause mortality before and after the implementation of the CS team. Secondary objectives included describing right heart catheterization (RHC) use, as well as MCS deployment and the selection of specific devices across both periods. Additionally, as an exploratory analysis, we aimed to describe our institutional experience with extracorporeal cardiopulmonary resuscitation (E-CPR), observed exclusively in the postimplementation period, including cardiac arrest characteristics and in-hospital outcomes.

### Data collection

Clinical information was obtained from the TecSalud Cardiogenic Shock Registry and institutional electronic medical records. The collected variables included demographic characteristics, clinical presentation, and invasive hemodynamics. Potential confounders and predictors included baseline demographic and hemodynamic characteristics, CS etiology, and—for the post-implementation cohort—SCAI stage, and laboratory markers. Shock phenotype and initial clinical severity were considered potential effect modifiers.

Because of the retrospective nature of the pre-CS team era and the lack of standardized hemodynamic and laboratory documentation in historical records, formal SCAI staging and comparative analysis of biochemical markers (such as lactate) were not possible for the preimplementation cohort. Baseline comparability was, instead, assessed using baseline demographics, admission vital signs, and the frequency of cardiac arrest. The intensity of clinical intervention, specifically the high utilization of MCS in the postimplementation era, was interpreted in the context of these baseline clinical anchors to assess disease acuity across both periods.

### Statistical analysis

Continuous variables were reported as mean ± SD or median (range), as appropriate, and categorical variables as counts and percentages. Mortality rates were analyzed using descriptive statistics and compared using the Fisher exact test. Missing data were handled using complete-case analysis. Variables with unavailable or incomplete documentation were not imputed and were excluded from the secondary analyses. Importantly, the primary outcome (30-day mortality) was available for all patients, allowing for complete inclusion in the main comparative analysis. Given the observational and retrospective nature of the study, the analysis was designed to evaluate associations rather than establish definitive causality. Furthermore, because of the small sample size and the limited number of primary events, formal multivariable regression modeling was not performed to avoid the risk of statistical overfitting. To mitigate potential bias, primary outcomes were strictly defined and validated across both eras. Statistical analyses were performed using RStudio version 4.5.1, with a 2-sided *P* <.05 considered statistically significant.

## Results

**Figure 2:**
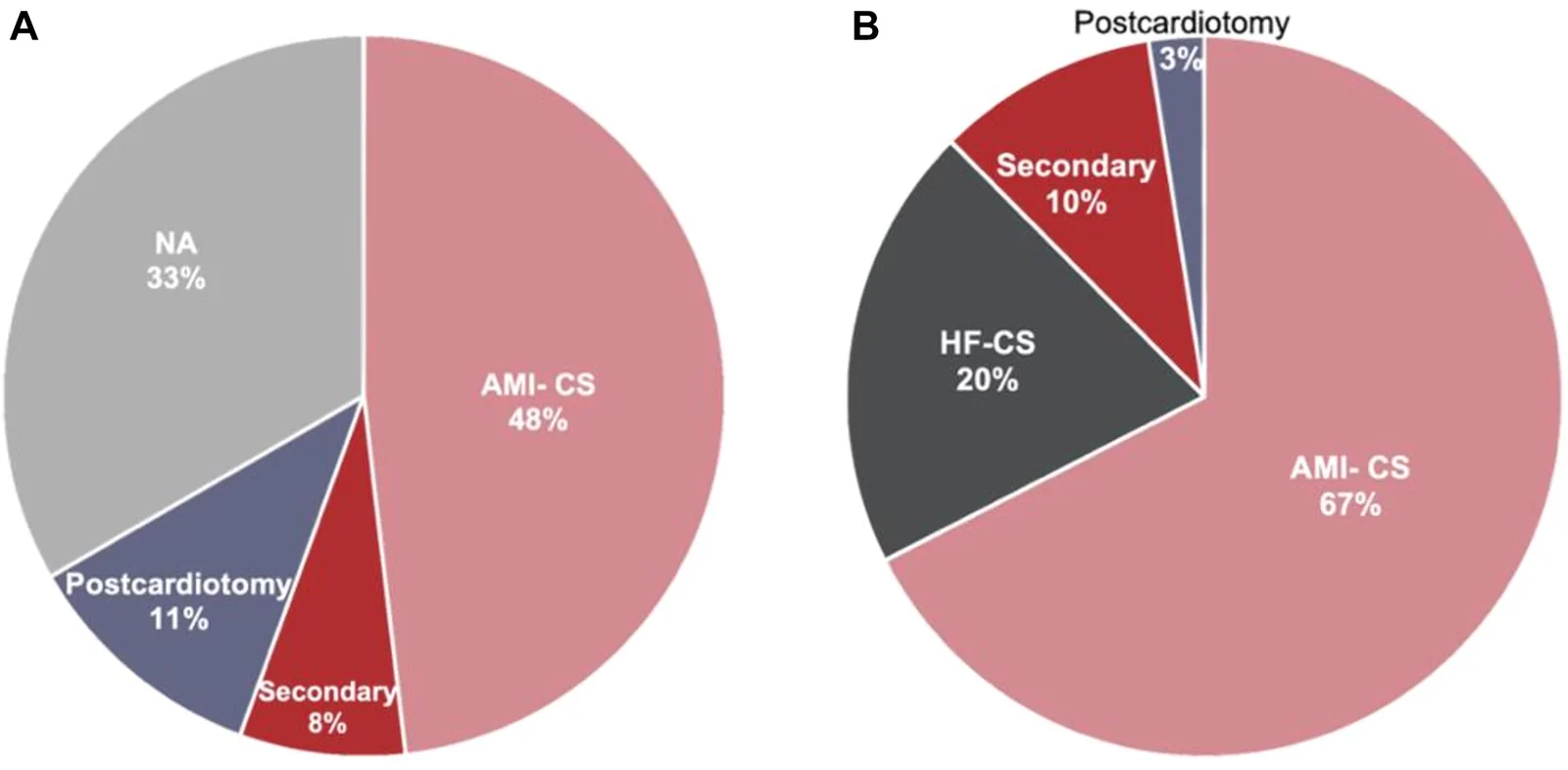
**Etiology of cardiogenic shock (CS) before and after Shock Team implementation.** Distribution of CS etiologies in the pre-CS team cohort (**A**) (n = 27) and the post-CS team cohort (**B**) (n = 40). AMI-CS, acute myocardial infarction–related CS; HF-CS, heart failure–related CS; NA, etiology not available.

A total of 67 patients with CS were included in the study and divided into 2 retrospective cohorts according to the multidisciplinary program implementation. The preteam cohort comprised 27 patients admitted between January 2021 and July 2022, before the establishment of the TecSalud Cardiogenic Shock Team. The posteam cohort included 40 patients admitted between August 2022 and August 2025, after the formal program implementation. Demographic and clinical parameters of both groups are described in Table 1. Notably, both populations present similar demographic characteristics. Prior to the formal implementation of the multidisciplinary shock program, standard-of-care practices lacked a protocolized approach to the systematic documentation of critical biochemical markers, such as serial arterial lactate, BNP, or arterial pH. Furthermore, CS etiology was better categorized after CS team instauration (Figure 2).

**Table 1:** Demographic, clinical, and management characteristics by CS study group.

	Pre-CS team (n = 27)	Post-CS team (n = 40)
Demographic variables		
Age, y	71 (39-83)	63.4 (21-88)
Women	6 (22)	11 (27)
Hospital LOS, d	9 (2-43)	11.5 (0-122)
ICU LOS, d	N/A	7 (1105)
BMI, kg/m^2^	26.1 (20-39) (n = 21)^a^	29.7 (20-37)
Smoking	8 (30)	30 (75)
Diabetes mellitus	9 (33)	19 (48)
Hypertension	13 (48)	17 (43)
CIHD	10 (37)	15 (38)
Atrial fibrillation	2 (7.4)	6 (15)
CVD	2 (7.4)	1 (2.5)
Heart failure	5 (19)	9 (23)
Clinical variables on admission		
SBP, mm Hg	105.5 (0-160)	105.5 (0-160)
DBP, mm Hg	60 (37-70)	61 (0-104)
MAP, mm Hg	67.5 (0-85)	75.5 (0-120)
HR, bpm	77 (56-130)	93.5 (0-160)
RR, breaths/min	20 (14-36)	18 (0-30)
O_2_ saturation, %	96 (60-100)	93 (0-99)
Temperature, °C	36.3 (36-36.9)	36.2 (35-38)
Cardiac arrest	16 (59)	17 (43)
Arrest time, min	N/A	32 (1-108) (n = 17)^a^
SCAI stage		
B	N/A	7 (18)
C	N/A	10 (25)
D	N/A	10 (25)
E	N/A	13 (33)
Shock phenotypes		
Right	N/A	6 (6)
Left	N/A	20 (50)
Biventricular	N/A	14 (35)
Shock characteristics		
Shock etiology		
AMI-CS	20 (60)	28 (70)
HF-CS	6 (20)	6 (15)
Other^b^	6 (20)	6 (15)
Troponin, ng/mL	N/A	463.5 (0-190,676)
BNP, pg/mL	N/A	391 (10-23,406) (n = 35)^a^
pH	N/A	7.32 (6.8-7.43)
Lactate, mmol/L	N/A	3.25 (0.7-15)
LVEF, %	N/A	34.5 (13-71) (n = 34)^a^
Management		
E-CPR	0 (0)	8 (20)
MCS	7 (26)	32 (80)
IABP	5 (19)	25 (63)
VA-ECMO	1 (4)	13 (33)
mAFP	1 (4)	8 (20)

Values are mean (range) (Age only), n (%), or median (range).

AMI, acute myocardial infarction; BMI, body mass index; BNP, B-type natriuretic peptide; CIHD, chronic ischemic heart disease; CS, cardiogenic shock; CVD, cerebrovascular disease; DBP, diastolic blood pressure; E-CPR, extracorporeal cardiopulmonary resuscitation; HF, heart failure; HR, heart rate; IABP, intra-aortic balloon pump; ICU, intensive care unit; LOS, length of stay; LVEF, left ventricular ejection fraction; mAFP, microaxial flow pump; MAP, mean arterial pressure; MCS, mechanical circulatory support; N/A, not available; RR, respiratory rate; SBP, systolic blood pressure; SCAI, Society for Cardiovascular Angiography & Interventions; VA-ECMO, venoarterial extracorporeal membrane oxygenation.

a Incomplete data; the number of patients analyzed varied according to the variable.

b Other CS etiologies (postcardiotomy/procedure related).

Although the total number of patients increased after implementation of the CS team, the annualized number of enrolled patients appears lower in the postteam period. This likely reflects differences in case identification and registry methodology, with the postimplementation era benefiting from a more structured and prospective data collection process.

### Primary end point

#### Mortality comparison

Following implementation of the TecSalud Cardiogenic Shock Team, a significant reduction in 30-day mortality was observed between the 2 study periods. Mortality decreased from 63% in the pre-CS team period to 25% in the post-CS team period (OR, 4.96; 95% CI, 1.57-16.87; *P* = .0025). This corresponds to nearly 5-fold higher odds of death before the program’s implementation. Notably, this improvement reflects an absolute risk reduction of nearly 40% that was sustained through mid-2025. The Central Illustration shows the comparative overall cumulative 30-day mortality rates before and after the team’s establishment.

**Central Illustration:**
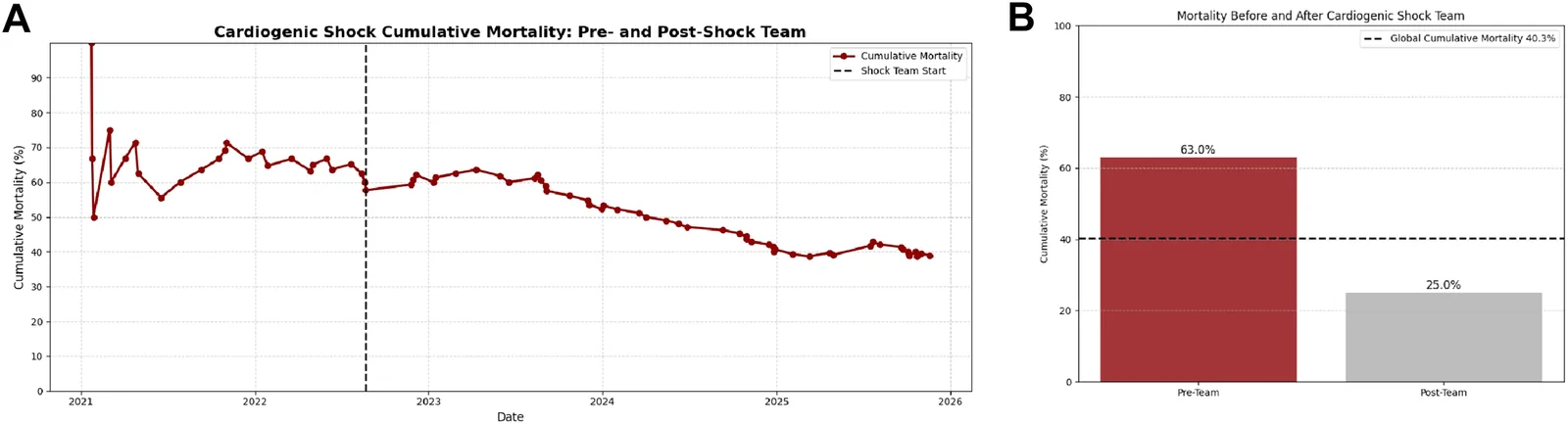
**Mortality comparison before and after cardiogenic Shock Team implementation.** (**A**) (Left) Temporal trend of cumulative mortality before and after the implementation of the cardiogenic Shock Team, showing a progressive decline following its initiation (black dashed line). (**B**) Comparison of cumulative mortality pre-implementation and post-implementation, demonstrating a reduction from 63.0% to 25.0%; the black line represents overall mortality (40.3%).

### Secondary end points

#### Right heart catheterization

Prior to the establishment of the institutional CS team, only 1 of 27 patients (4%) underwent RHC. Following implementation, the rate increased markedly to 28 of 40 patients (70%), reflecting a significant increase in the use of invasive hemodynamic monitoring as part of the standardized Shock Team protocol.

#### Mechanical circulatory support

**Figure 3:**
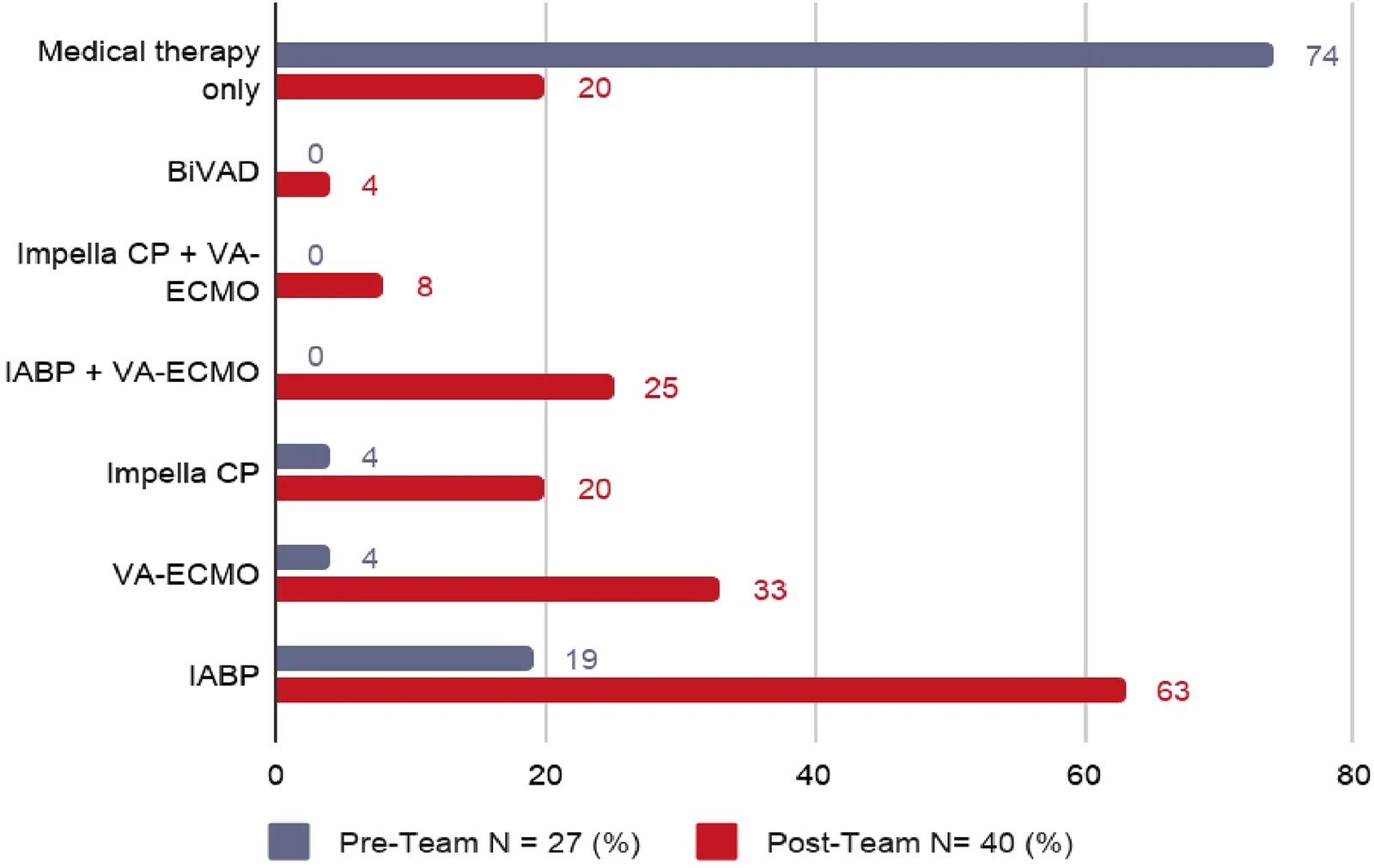
**Utilization of MCS modalities before and after Shock Team implementation.** Bars represent the percentage of patients receiving each modality in the preteam and postteam periods (preteam n = 27; postteam n = 40). BiVAD, biventricular assist device; IABP, intraaortic balloon pump; VA-ECMO, venoarterial extracorporeal membrane oxygenation.

In the preteam period, 7 of 27 patients (26%) received some form of MCS. Intraaortic balloon pump (IABP) was most used (19%), followed by ECMO and Impella (each 4%). After team implementation, MCS was employed in 32 of 40 patients (80%), demonstrating a major shift toward earlier and broader integration of advanced hemodynamic support within the structured management pathway. IABP remained the most frequently used modality (63%), followed by venoarterial extracorporeal membrane oxygenation (VA-ECMO) (33%) and Impella CP (20%). Combination strategies were employed in a subset of patients, including IABP + VA-ECMO in 25%, Impella CP + VA-ECMO in 8%, and biventricular assist device configuration in 1 patient (Figure 3).

#### E-CPR

A total of 17 patients (43%) experienced in-hospital cardiac arrest, with a median arrest duration of 32 minutes. Among them, 7 patients (41%) underwent E-CPR. The median no-flow and low-flow times were 1.4 and 41 minutes, respectively, and all patients achieved return of spontaneous circulation. The most common etiology was acute myocardial infarction–related CS (3 patients, 42.8%). In-hospital mortality among patients treated with E-CPR was 57.4%.

## Discussion

The development of a structured CS program in Latin America required several fundamental components: a multidisciplinary framework, standardized protocols, comprehensive education and training, and a continuous process of quality assessment and improvement. Establishing such a program at the flagship hospital of TecSalud presented significant clinical, logistical, and organizational challenges inherent to our local health care environment. Despite these challenges, unwavering physician leadership, coupled with persistence and institutional support, enabled the program's successful implementation. Our experience demonstrates that even in complex health care environments with regional resource disparities, coordinated, protocol-based multidisciplinary care can markedly improve outcomes in patients with CS.

### Local context and baseline challenges

Before the implementation of TecSalud’s CS program, in-hospital mortality among patients with CS was alarmingly high, reaching 63% at 30 days. This rate is consistent with published data from the United States showing that short-term mortality frequently exceeds 50%, despite advances in revascularization and supportive therapy.^1^ However, it surpasses the mortality reported in pivotal structured intervention trials such as SHOCK, IABP-SHOCK II, and CULPRIT-SHOCK, where 30-day mortality ranged from 40% to 50%.^6^, ^7^, ^8^

In the pre-CS team era, multiple factors contributed to these unfavorable outcomes. Fragmented care delivery, limited availability of MCS devices, and inertia toward change contributed to delayed recognition and intervention for patients in CS. Awareness of CS as a distinct, time-sensitive syndrome requiring coordinated management was low among health care providers, resulting in delayed decision-making and inconsistent treatment pathways. These challenges mirror those observed across Latin America, where organized CS programs remain rare and national data are scarce.^9^^,^^10^ Moreover, broader systemic constraints—including underfunded public health systems, disparities in access to advanced technologies, and shortages of specialized personnel—remain prevalent in the region. These structural barriers present a stark contrast to the private sector context in which our institution operates.

### The multidisciplinary “Shock Team” model

The TecSalud CS multidisciplinary team included interventional cardiologists, advanced heart failure specialists, cardiac surgeons, critical care physicians, ECMO specialists, cardiac imaging experts, nurses, and administrative staff. Each member had clearly defined roles and responsibilities within an integrated communication structure. Early team activation enabled prompt hemodynamic evaluation, revascularization when indicated, and timely MCS initiation. Previous studies have demonstrated that early multidisciplinary team involvement correlates with improved CS survival.^3^^,^^11^^,^^12^ TecSalud’s experience supports these findings, underscoring the necessity of cross-disciplinary coordination in managing this complex syndrome.

### Education and capacity building

Education and sustained training were critical to program success. A structured curriculum was developed to enhance recognition and management of CS across all levels of care. Training included both theoretical and hands-on components: hemodynamic interpretation, MCS device management (IABP, Impella CP, and ECMO), and interprofessional communication. Simulation-based exercises allowed teams to rehearse high-acuity scenarios, improving coordination under pressure. Educational sessions targeted not only cardiologists and fellows but also emergency physicians, intensivists, and nursing staff to ensure institutional readiness.

Ongoing engagement with international experts through webinars and collaborative case discussions reinforced adherence to global best practices and ensured alignment with evolving consensus recommendations.^13^^,^^14^ This emphasis on capacity building was essential for sustaining program quality and promoting the teamwork culture that underpins effective CS management.

### Protocol development and implementation

The implementation phase prioritized rapid identification of CS and early activation of the multidisciplinary team. The “SUSPECT CS” mnemonic has been incorporated into daily clinical practice to improve bedside recognition,^1^^,^^15^ encompassing key clinical domains including signs of hypoperfusion, hemodynamic instability, escalating vasopressor requirements, and objective evidence of cardiac dysfunction or congestion. Diagnostic algorithms included serial biomarker assessment (high-sensitivity troponin, NT-proBNP, D-dimer), metabolic and hepatic panels, and focused echocardiography to evaluate ventricular function.^16^ When appropriate, RHC was employed for hemodynamic profiling, incorporating indices such as cardiac power output and pulmonary artery pulsatility index, which refine risk stratification and guide MCS selection.^17^

To streamline workflow, TecSalud instituted a “Code Shock” system providing 24/7/365 coverage through dedicated weekly rotating teams. Activation can be triggered from any clinical setting, including the emergency department, intensive care unit, catheterization laboratory, or general wards, beginning with an initial evaluation by the first-contact physician followed by rapid consultation with the CS team to verify activation criteria. This system promotes synchronized action among cardiology, surgery, critical care, and perfusion services, ensuring that all stakeholders operate within a unified algorithm. Crucially, the CS team maintains collaborative links with other institutional response units, such as the pulmonary embolism and ST-elevation myocardial infarction teams. Once active, treatment strategies emphasize minimizing vasopressor use, prioritizing myocardial recovery, and tailoring interventions to the specific CS phenotype and local resource availability.

### Cost considerations and health system context

The increased utilization of MCS following the implementation of the CS team has important economic implications. In our institution, which operates within a private, university-based, not-for-profit health care system, the costs associated with advanced MCS were generally borne through a private insurance authorization and institutional administrative support. The establishment of the multidisciplinary CS team facilitated not only clinical decision-making but also the rapid alignment of administrative and financial processes, allowing timely approval of resource-intensive therapies in critically ill patients. Importantly, this model reflects a unique institutional framework and may not be generalizable to public sector hospitals or other resource-limited settings where access to MCS remains constrained by financial and infrastructural barriers.

### Device selection and MCS strategy

The selection of MCS modality was not restricted by a predefined annual case volume or device-specific quota. Instead, decisions were guided by a multidisciplinary assessment. Additionally, real-time payor authorization and institutional resource availability were considered in parallel to clinical urgency. Impella CP use was, therefore, individualized and employed selectively in cases where left ventricular unloading was deemed beneficial, either as a standalone device or in combination with VA-ECMO.

### Continuous improvement and debriefing

A structured postcase debriefing process was integral to program maturation. The multidisciplinary team reviewed each case to evaluate clinical decisions, procedural performance, and communication dynamics. This iterative process identified areas for improvement, fostered accountability, and informed protocol revisions. By promoting open dialog and shared learning, debriefings transformed individual experiences into institutional knowledge, reinforcing a culture of continuous improvement.

### Outcomes and impact

Following its implementation, TecSalud achieved a remarkable reduction in 30-day mortality from 63% to 25% (38% absolute and 60% relative risk reduction), closely aligning with outcomes from established networks such as the Detroit Cardiogenic Shock Initiative (25%-35%).^18^ However, it is important to acknowledge that our primary end point carries a wide CI (95% CI, 1.57-16.87). This broad range highlights the limitations in precision inherent to our sample size, meaning that although the survival trend is strongly positive, the true effect size may vary.

The driver of this success extended beyond simply assembling a group of experts; a fundamental structural shift was required to overcome the fragmentation inherent in the private “open” practice model. This success was predicated on the alignment of institutional stakeholders and the optimization of administrative pathways, which allowed the CS team to act as a vital link bridging the gap between technological availability and clinical urgency. By streamlining internal coordination and institutional workflows, the team eliminated traditional logistical barriers, ensuring that high-intensity interventions were delivered without the delays typical of a nonprotocolized private system. In the preteam period, MCS use was low (26%) and relied heavily on IABP, reflecting a lack of unified decision-making. The dramatic increase in MCS utilization (80%) in the postimplementation period was not merely a resource upgrade, but the result of centralizing clinical governance. Some multicenter studies, such as those from the Cardiac Critical Care Trials Network,^3^ have reported a stabilization or even a decrease in overall MCS use following Shock Team implementation; our experience demonstrated a significant increase. This discrepancy likely reflects our baseline “preteam” era's reliance on fragmented care, where MCS was underutilized because of the lack of a standardized escalation protocol. Furthermore, the shift was not just in quantity but in quality; the postimplementation period saw a move away from routine IABP toward more robust hemodynamic support platforms tailored to the patient’s SCAI stage and phenotype. In our setting, the Shock Team acted as a catalyst for appropriate escalation in patients who previously would have been managed with medical therapy alone despite failing hemodynamics. A central question is whether the observed survival benefit is primarily driven by the multidisciplinary team structure or the increased utilization of advanced MCS; we contend that these 2 factors are functionally interdependent. The multidisciplinary team provided the essential clinical governance required to not only increase the use of advanced therapies but to optimize their timing, selection, and complication management. Without a protocolized “Code Shock” to ensure rapid, coordinated decision-making, the escalation to high-intensity MCS platforms would likely have been fragmented and less effective. Therefore, the mortality reduction represents a synergistic effect where the team structure serves as the decision-making framework that enables the safe and targeted application of MCS. This integration was paramount in achieving survival rates comparable to international benchmarks. The cohort size (n = 40) reflects these initial challenges of implementation within an open private practice model, yet it simultaneously represents the successful transition from reactive rescue therapy to a proactive recognition system. This transition is evidenced by the shift in clinical presentation between the 2 eras; the higher frequency of cardiac arrest in the preteam era (59% vs 43%) likely reflects a system that historically recognized shock only at its terminal stages. After implementing the “Code Shock” and increasing institutional awareness, we were able to intercept patients earlier in their clinical course—often before the onset of cardiac arrest—allowing for timely escalation to advanced MCS. By establishing a single, protocol-driven authority that superseded individual physician preferences, we ensured timely, standardized escalation. These findings suggest that in the Latin American private sector, success depends on dismantling the silos of independent practice to create a cohesive, academic-style response system comparable to high-income benchmarks. Furthermore, although our center’s access to advanced technology is a notable factor, the successful implementation of the program was largely dependent on overcoming the socioeconomic barriers inherent to the region. By establishing institutional frameworks that aligned clinical need with administrative feasibility, we were able to provide high-intensity care that is often hindered by financial and logistical hurdles in LMIC. This model suggests that the “Shock Team” acts not only as a clinical unit but as an organizational solution for resource optimization in complex health care environments.

### Regional collaboration and knowledge dissemination

Beyond institutional success, TecSalud has prioritized regional collaboration to advance CS care in Latin America. The organization of the first Latin American Symposium on Cardiogenic Shock established a platform for interinstitutional dialog, data sharing, and cooperative education. Such efforts aim to develop a network of excellence that standardizes care pathways and supports the broader adoption of multidisciplinary shock teams across the region. Strengthening regional collaboration is essential to bridging disparities in access, fostering innovation, and elevating the standard of care for patients with CS throughout Latin America.

### Limitations

This study has several important limitations. First, its retrospective, single-center design inherently limits the ability to establish a direct causal relationship between the CS team implementation and the observed mortality reduction. Although the association is strong, other unmeasured temporal factors—such as evolving institutional expertise, improvements in general intensive care unit care, or shifts in patient selection—may have contributed to the outcomes. However, it is noteworthy that many of these institutional improvements were directly catalyzed by the CS program, which fostered a multidisciplinary culture of early recognition and streamlined coordination that permeated the broader cardiovascular service line. Nevertheless, as the study spans a 5-year period, we cannot entirely isolate the program’s specific effects from broader secular trends in cardiovascular care during this time.

Furthermore, the absence of prospective SCAI staging and comparative laboratory markers for the preimplementation cohort is a significant constraint. Although we utilized admission vital signs and the frequency of cardiac arrest as clinical anchors to assess baseline acuity, the lack of granular hemodynamic data prevents a definitive, matched comparison of shock severity between the 2 eras. This introduces a risk of selection bias, as we cannot statistically exclude the possibility that baseline differences influenced the outcomes.

The relatively small sample size is a notable limitation that may impact the statistical stability of our findings and potentially lead to an overestimation of the effect size. Although the mortality reduction of nearly 40% is highly encouraging, the lack of multivariable adjustment prevents us from ruling out residual confounding. Therefore, our results should be interpreted as a promising association with the instauration of the multidisciplinary program, rather than a definitive cause. Given the sample size and the frequent use of combined MCS strategies, outcomes stratified by specific MCS modality and SCAI stages are presented descriptively, without formal statistical comparison, and should be interpreted with caution. Furthermore, the limited number of primary events precluded the use of robust multivariable modeling to adjust for potential confounders such as age, shock etiology, or SCAI stage; therefore, the potential for residual confounding remains, and our findings may carry a risk of overestimating a true effect size.

In addition, our institution operates within the private health care sector, where resource availability, care pathways, and patient profiles may differ significantly from those in public systems, potentially limiting the applicability of our findings to the broader regional context. Specifically, the high rate of MCS utilization observed in our cohort was facilitated by unique institutional and administrative alignments that may not be readily available in underfunded public health systems or other LMIC with more extreme resource constraints. Therefore, the generalizability of our mortality outcomes to centers without similar access to advanced circulatory support or the institutional capacity for rapid multidisciplinary activation may be restricted.

Although preimplementation data were available, not all baseline demographic and clinical variables were consistently recorded in the pre-CS team cohort. Analyses were restricted to variables with sufficient completeness, precluding fully adjusted comparisons between periods and introducing the potential for residual confounding.

Temporal changes, specifically, secular trends in critical care—such as evolving referral patterns, the learning curve associated with new protocols, and the increased availability and familiarity with MCS devices over time—may have contributed to the improved outcomes. Furthermore, because this study evaluates a local, single-center program, our results may not be directly generalizable to other institutions with different health care infrastructures. Finally, although longitudinal follow-up is ongoing, the current analysis is limited to short-term outcomes, as longer-term data are still being collected and were not included in this report.

## Conclusion

The implementation of a multidisciplinary CS program at a tertiary center in Mexico was associated with a significant reduction in short-term mortality. These findings demonstrate the feasibility and potential impact of protocolized care even in complex health care environments where financial and systemic barriers often hinder access to advanced therapies. This program illustrates how a coordinated, team-based strategy can successfully navigate the complexities of the private “open” practice model through unique institutional agreements, effectively narrowing regional care gaps. Although causal inference is limited by the study design, this program serves as a promising, scalable model for strengthening CS management across Latin America by achieving outcomes comparable to those of high-income regions despite prevailing socioeconomic constraints.

## CRediT authorship contribution statement

**Vicente Jimenez-Franco:** Conceptualization, Data curation, Methodology, Project administration, Writing – original draft, Writing – review & editing. **Jahir Rodriguez-Rivera:** Conceptualization, Project administration, Writing – original draft, Writing – review & editing. **Renata Quevedo-Salazar:** Conceptualization, Formal analysis, Project administration, Writing – original draft, Writing – review & editing. **Jose A. Salinas-Casanova:** Conceptualization, Data curation, Investigation, Writing – original draft, Writing – review & editing. **Monica Flores-Zertuche:** Conceptualization, Writing – original draft, Writing – review & editing. **Cecilia Gocher-Janet:** Conceptualization, Investigation, Writing – original draft, Writing – review & editing. **Juan Carlos Ibarrola-Peña:** Conceptualization, Investigation, Writing – original draft. **Carlos Jerjes-Sanchez:** Conceptualization, Funding acquisition, Project administration, Supervision, Validation, Writing – original draft, Writing – review & editing. **Rene D. Gomez-Gutierrez:** Conceptualization, Project administration, Resources, Validation, Visualization, Writing – original draft. **Guillermo Quezada-Valenzuela:** Conceptualization, Validation, Writing – original draft. **Erasmo de la Peña:** Conceptualization, Investigation, Project administration, Validation, Writing – original draft, Writing – review & editing. **Guillermo Torre-Amione:** Conceptualization, Investigation, Supervision, Validation, Visualization, Writing – original draft, Writing – review & editing.

## Declaration of competing interest

The authors declared no potential conflicts of interest with respect to the research, authorship, and/or publication of this article.
